# A three-microRNA signature for lung squamous cell carcinoma diagnosis in Chinese male patients

**DOI:** 10.18632/oncotarget.19666

**Published:** 2017-07-28

**Authors:** Lan Zhang, Xia Shan, Jun Wang, Jun Zhu, Zebo Huang, Huo Zhang, Xin Zhou, Wenfang Cheng, Yongqian Shu, Wei Zhu, Ping Liu

**Affiliations:** ^1^ Department of Oncology, First Affiliated Hospital of Nanjing Medical University, Nanjing 210029, PR China; ^2^ Department of Respiration, The Affiliated Jiangning Hospital of Nanjing Medical University, Nanjing 210000, PR China; ^3^ Department of Thoracic Surgery, First Affiliated Hospital of Nanjing Medical University, Nanjing 210029, PR China; ^4^ Department of Radiation Oncology, Jiangsu Cancer Hospital, Nanjing 210009, PR China; ^5^ Department of Gastroenterology, First Affiliated Hospital of Nanjing Medical University, Nanjing 210029, PR China

**Keywords:** serum microRNA, lung SCC, diagnostic biomarker, qRT-PCR

## Abstract

Various studies have demonstrated the diagnostic value of microRNA (miRNA) for lung cancer, but miRNA signatures varied between different subtypes. Whether serum miRNAs could be used as biomarkers in lung squamous cell carcinoma (SCC) remains unknown. Using quantitative real-time polymerase chain reaction (qRT-PCR) based Exiqon panel, 38 differentially expressed miRNAs were identified from 3 male lung SCC pool samples and 1 normal control (NC) pool in the initial screening phase. After the training (24 SCC VS. 15 NCs), testing (44 SCC VS. 57 NCs) and external validation (34 SCC VS. 36 NCs VS. 10 pulmonary hamartoma) processes via qRT-PCR, we identified a three-miRNA panel ((miR-106a-5p, miR-20a-5p and miR-93-5p) to be a potential diagnostic marker for male lung SCC patients. The areas under the receiver operating characteristic (ROC) curve of the three-miRNA panel for the training, testing and validation phases were 0.969, 0.881 and 0.954 respectively. In addition, this signature could also differentiate lung SCC from pulmonary hamartoma (AUC=0.900). The 3 miRNAs were consistently up-regulated in lung SCC tissues (23 SCC VS. 23 NCs) and serum exosomes (17 SCC VS. 24 NCs). Moreover, expression of the 3 miRNAs was decreased in arterial serum (n = 3). In conclusion, we established a three-miRNA signature in the peripheral serum with considerable clinical value in the diagnosis of male lung SCC patients.

## INTRODUCTION

Lung cancer is the leading cause of cancer mortality in both men and women worldwide [1]. Non-small cell lung cancer (NSCLC) accounts for approximately 85% of all lung cancers. Among NSCLCs, squamous cell carcinoma (SCC) is one of the major histological subtypes [2]. The majority of lung SCC patients is male and had a smoking history [3, 4]. Although surgical resection shows promise in treating lung SCC, a lack of effective tools for early diagnose leads to low 5-year survival rates [2]. Low-dose computed tomography (LDCT) has been explored as a method for the diagnosis of early lung SCC, however, it still has several limitations [5]. None-invasive markers such as squamous cell carcinoma antigen (SCC Ag), neuron-specific enolase (NSE) and Cyfra 21-1 are not sensitive and specific enough for the routine use in the diagnose of lung SCC [6]. Thus, new and non-invasive biomarkers with high diagnostic power for lung SCC patients are urgently needed.

Circulating miRNAs have emerged as reliable non-invasive biomarkers for the early diagnosis of cancer [7, 8]. Specifically, the expression patterns of human serum miRNAs have been reported to have the potential to identify various types of cancers [9–12]. These findings suggest that there might also be a unique serum miRNA expression signature that could distinguish lung SCC patients with normal individuals. Male accounts for more than 90% of lung SCC patients and have different biological behavior and treatment response compared with female patients [2]. So it is of great significance to discover a unique miRNA signature for the diagnosis of male lung SCC patients. However, unlike the assessment of cellular miRNA levels for which there are accepted housekeeping genes, it still remains unclear whether any circulating miRNAs have prototypical housekeeping functions or could be present at sufficiently stable levels to serve as effective reference controls.

In the present study, using both miRCURY platform and quantitative real-time polymerase chain reaction (qRT-PCR), we sought to identify a panel of serum miRNAs that could serve as a novel biomarker for the diagnosis of male lung SCC patients. The expression profile of selected miRNAs was then assessed in tissue and serum exosomes to explore the origin and potential form in circulation. Finally, we compared miRNA expression in peripheral venous and arterial serum.

## RESULTS

### Identification of stable reference miRNAs in human serum samples

The potential reference miRNAs (miR-16-5p [13, 14], miR-103a-3p [15, 16], U6 [11, 16] and miR-191-5p [11, 17]) examined in present study were selected primarily because they had relatively stable expression level according to previous studies. In geNorm, high variation in expression elevates M values and indicates low stability, whereas low M values indicate high expression stability [16, 18]. In human serum samples, miR-16-5p and miR-103a-3p both showed the lowest M value (Supplementary Figure 1). In addition, miR-16-5p might be more stable than miR-103a-3p because of the lower Ct value compared with miR-103a-3p (Supplementary Table 2). Therefore, miR-16-5p was selected as the internal reference control in the present study.

### Patient description

**Figure 1:**
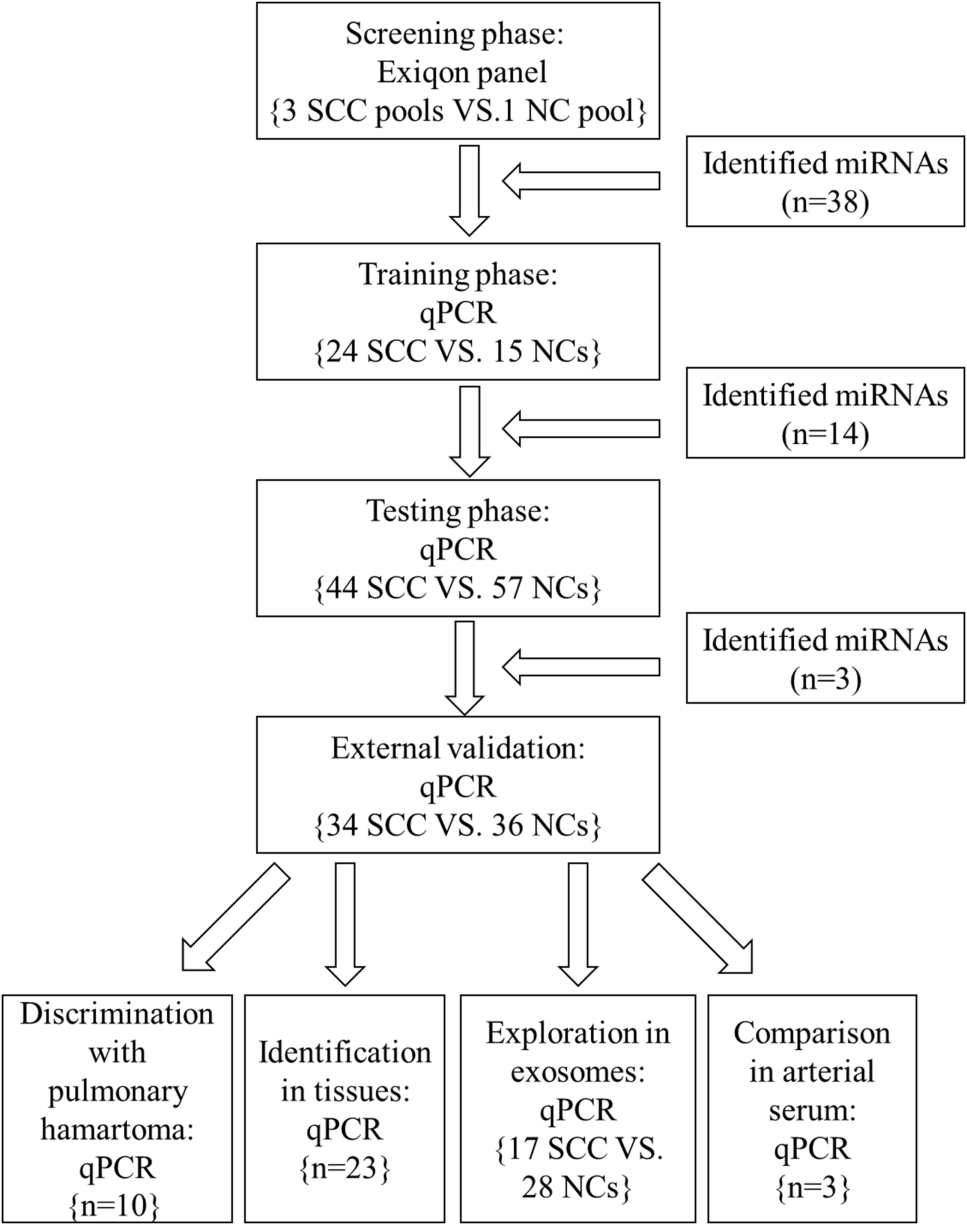
The flow chart of the experiment design SCC: squamous cell carcinoma; NC: normal control.

Serum samples from male lung SCC patients and NCs were randomly assigned to 3 independent sets: training stage, testing stage and external validation stage (the flow chart was shown in Figure 1). The clinical and demographics features of the lung SCC patients and NCs are listed in Table 1. There were no significant differences in the distribution of age, smoking and sex between the cancer patients and NCs.

**Table 1:** Characteristics of 102 SCC patients and 108 normal controls enrolled in the study

Variables	Screening phase (n=40)		Training stage (n=39)		Testing stage (n=101)		External validation stage (n=70)	
	Case (%)	Controls (%)	Case (%)	Controls (%)	Case (%)	Controls (%)	Case (%)	Controls (%)
Number	30	10	24	15	44	57	34	36
Age								
< 65	19 (63.3)	6 (60.0)	15 (62.5)	9 (60.0)	25 (56.8)	32 (56.1)	20 (58.8)	19 (52.8)
≥ 65	11 (36.7)	4 (40.0)	9 (37.5)	6 (40.0)	19 (43.2)	25 (43.9)	14 (41.2)	17 (47.2)
Smoking history								
Former	6 (20.0)	3 (30.0)	3 (12.5)	2 (13.3)	5 (11.3)	8 (14.0)	3 (8.80)	3 (8.80)
Current	24 (80.0)	7 (70.0)	21 (87.5)	13 (86.7)	39 (88.7)	49 (86.0)	31 (91.2)	33 (91.2)
TNM stage								
I	6 (20.0)		8 (33.3)		18 (40.9)		14 (41.2)	
II	17 (56.7)		13 (54.2)		21 (47.7)		12 (35.3)	
III	7 (23.3)		3 (12.5)		5 (11.4)		8 (23.5)	
Differentiation								
Well	3 (10.0)		3 (12.5)		5 (11.4)		3 (8.80)	
Moderately	18 (60.0)		12 (50.0)		21 (47.7)		18 (52.9)	
Poorly	9 (30.0)		9 (37.5)		18 (40.9)		13 (38.3)	

### Discovery of candidate miRNAs from pooled serum samples

A total of 168 miRNAs were sequenced by the Exiqon miRCURY-Ready-to-Use PCR-Human-panel-I+II-V1.M based on the qRT-PCR platform between in 3 peripheral serum pools from 30 male lung SCC patients and 1 pooled sample from 10 NCs. Candidate miRNAs were selected based on the following criteria: (a) with a Ct value < 37; (b) exhibiting 5 Ct lower than negative control (No Template Control, NTC); (c) having at least a 1.5-fold altered expression. Levels of 26 miRNAs were significantly higher in male lung SCC patients than controls. In contrast, levels of 12 miRNAs were significantly lower (Supplementary Table 1). Taken together, 38 differentially expressed miRNAs were selected as candidate miRNAs and were chosen to be further assessed via qRT-PCR.

### Confirmation of candidate miRNAs by qRT-PCR analysis

**Figure 2:**
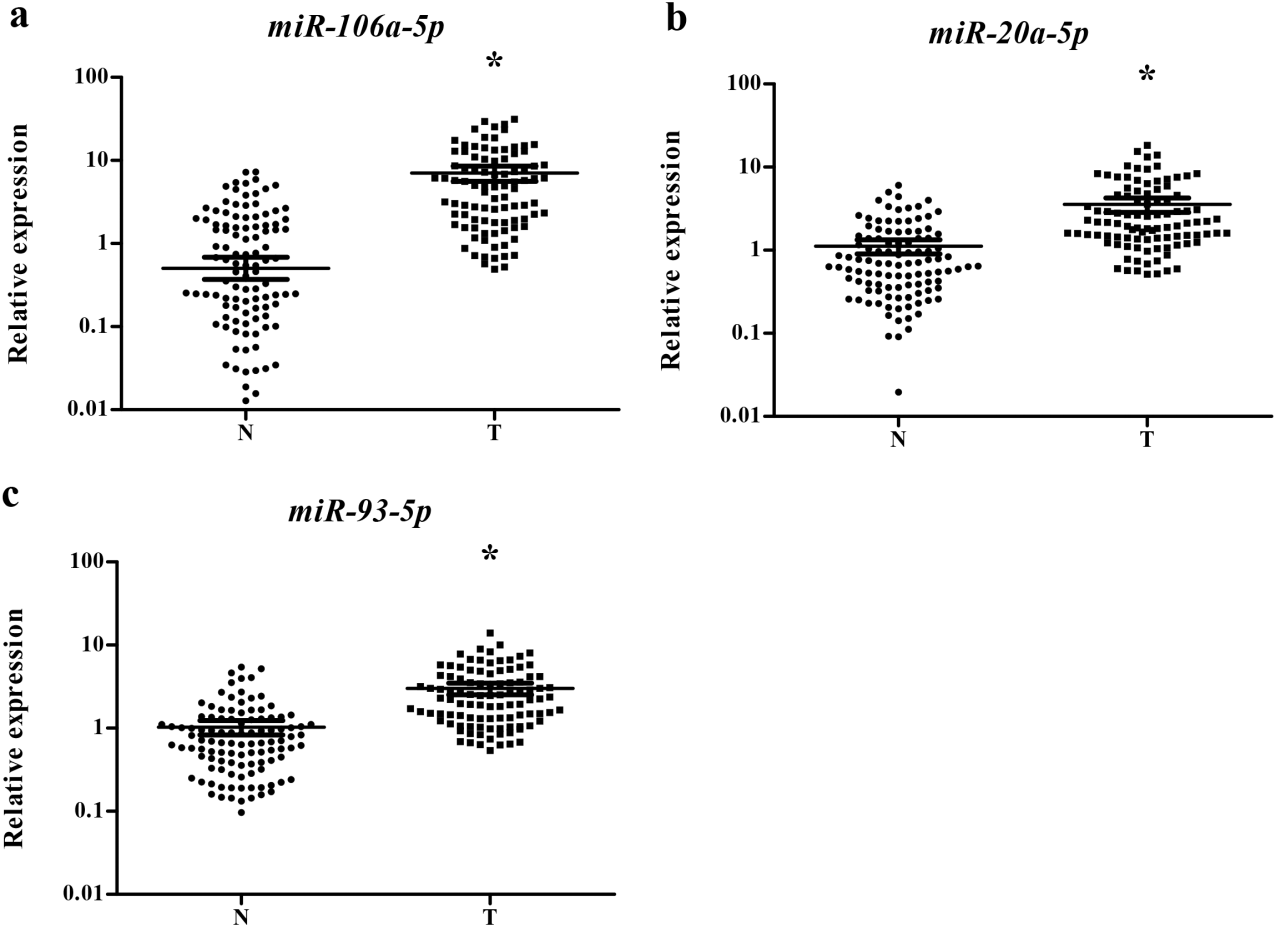
Expression levels of the three miRNAs in the serum of 102 lung SCC patients and 108 NCs **(a)** miR-106a-5p; **(b)** miR-20a-5p; **(c)** miR-93-5p; N: normal controls; T: tumor. Horizontal line: mean with 95% CI. *: P < 0.001.

Our study first tested the 38 candidate miRNAs in the training stage including 24 male lung SCC patients and 15 NCs. A total of 14 miRNAs demonstrated differential expression and were validate in the testing stage (Supplementary Table 1). In the larger cohort, 3 of the 14 miRNAs (miR-106a-5p, miR-20a-5p and miR-93-5p) showed consistent up-regulation. To verify the three miRNAs as a signature for male lung SCC patients, the expression of the three miRNAs was further assessed using an additional independent cohort of 34 lung SCC patients and 36 NCs. The expression trend of the three miRNAs was consistent with the training set and the testing stages, with all the miRNAs were upregulated > 2-fold (Supplementary Table 2). As a result of our multiphase analysis, we identified a three-miRNA panel to be the potential signature for the detection of male lung SCC patients (Figure 2).

### Diagnostic value of the three-miRNA signature in serum

**Figure 3:**
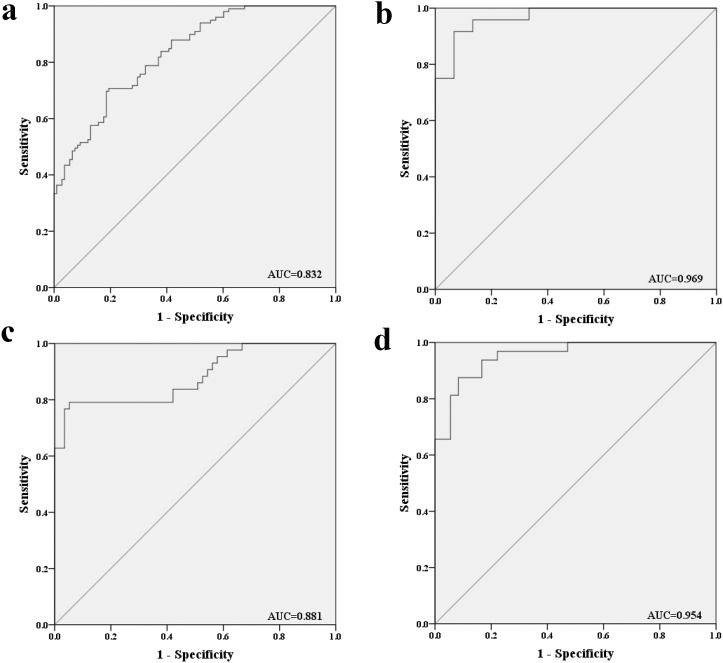
Receiver-operating characteristic (ROC) curves for the three-miRNA panel to discriminate lung SCC patients from NCs **(a)** the combined three phases of training, testing and external validation phases (102 SCC VS. 108 NCs); **(b)** training phase (24 SCC VS. 15 NCs); **(c)** testing phase (44 SCC VS. 57 NCs). **(d)** external validation (34 SCC VS. 36 NCs). AUC: areas under the curve.

To evaluate the diagnostic value of this three-miRNA signature in discriminating male lung SCC patients from NCs, ROC curve analysis was performed. The AUCs were 0.834 (95% confidence interval (CI):0.781-0.887), 0.804 (95% CI:0.746-0.863), 0.823 (95% CI:0.767-0.879) respectively (Supplementary Figure 2). Moreover, when the three miRNAs were combined together as a panel, the AUCs were 0.832 (95% CI: 0.780-0.885; Figure 3a). Meanwhile, the diagnostic performance of the three-miRNA panel was also assessed in the training, testing and external validation stages separately and the AUCs were 0.969 (95% CI: 0.924–1.000; Figure 3b), 0.881 (95% CI: 0.810–0.953; Figure 3c) and 0.954 (95% CI: 0.910–0.998; Figure 3d) respectively.

In addition, the diagnostic performance of the established miRNA panel was also analyzed at different TNM stage. The corresponding AUCs for lung SCC patients with TNM stage I, II, III were 0.904, 0.862 and 0.882 respectively (Supplementary Figure 3a-3c).

Our study also evaluated the diagnostic value of the three-miRNA panel in discriminating lung SCC from pulmonary hamartoma in the external validation stage. The result showed that the miRNA panel possessed high accuracy in discriminating lung SCC from pulmonary hamartoma (AUC=0.900; 95% CI: 0.804-0.996; Supplementary Figure 3d).

Finally, the association of the three serum miRNAs with clinical stage was also evaluated. However, none of the three miRNAs demonstrated significant difference in patients with stage III compared to those with stage I+II (data not shown).

### Identification of miRNA expression in tissue samples

**Figure 4:**
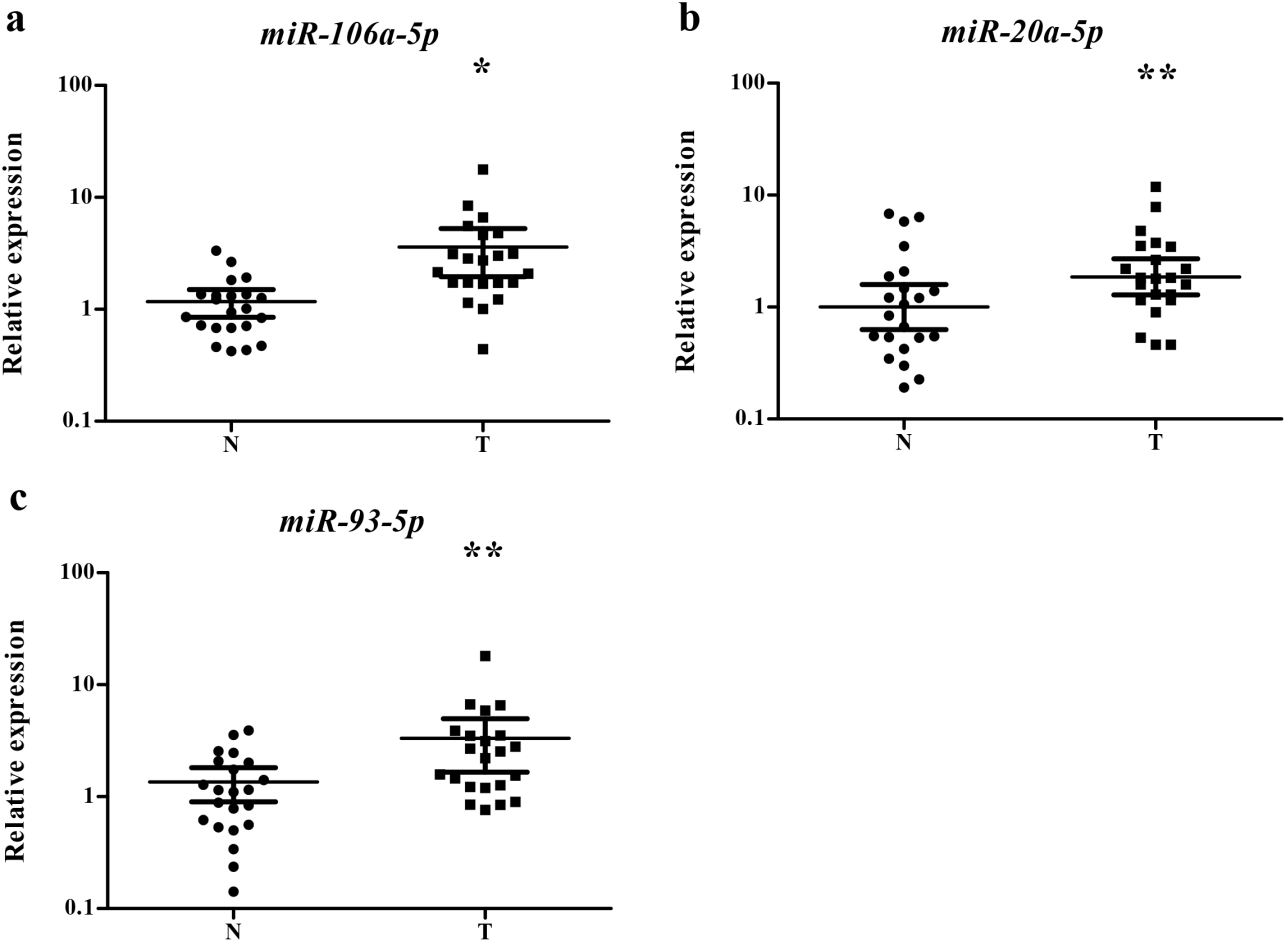
Expression of the three miRNAs in the tumor tissues of 23 pairs of lung SCC patients **(a)** miR-106a-5p; **(b)** miR-20a-5p; **(c)** miR-93-5p; N: normal controls; T: tumor. Horizontal line: mean with 95% CI. *: P < 0.001; **:P < 0.05.

The expression levels of the three miRNAs were examined in 23 pairs of tissue samples of lung SCC patients. As it is demonstrated in Figure 4, the expression of miR-106a-5p, miR-20a-5p and miR-93-5p was significantly higher in tumor tissue samples than the NCs, which was consistent with the result obtained from serum samples.

### Exploration of miRNA expression in serum exosomes

**Figure 5:**
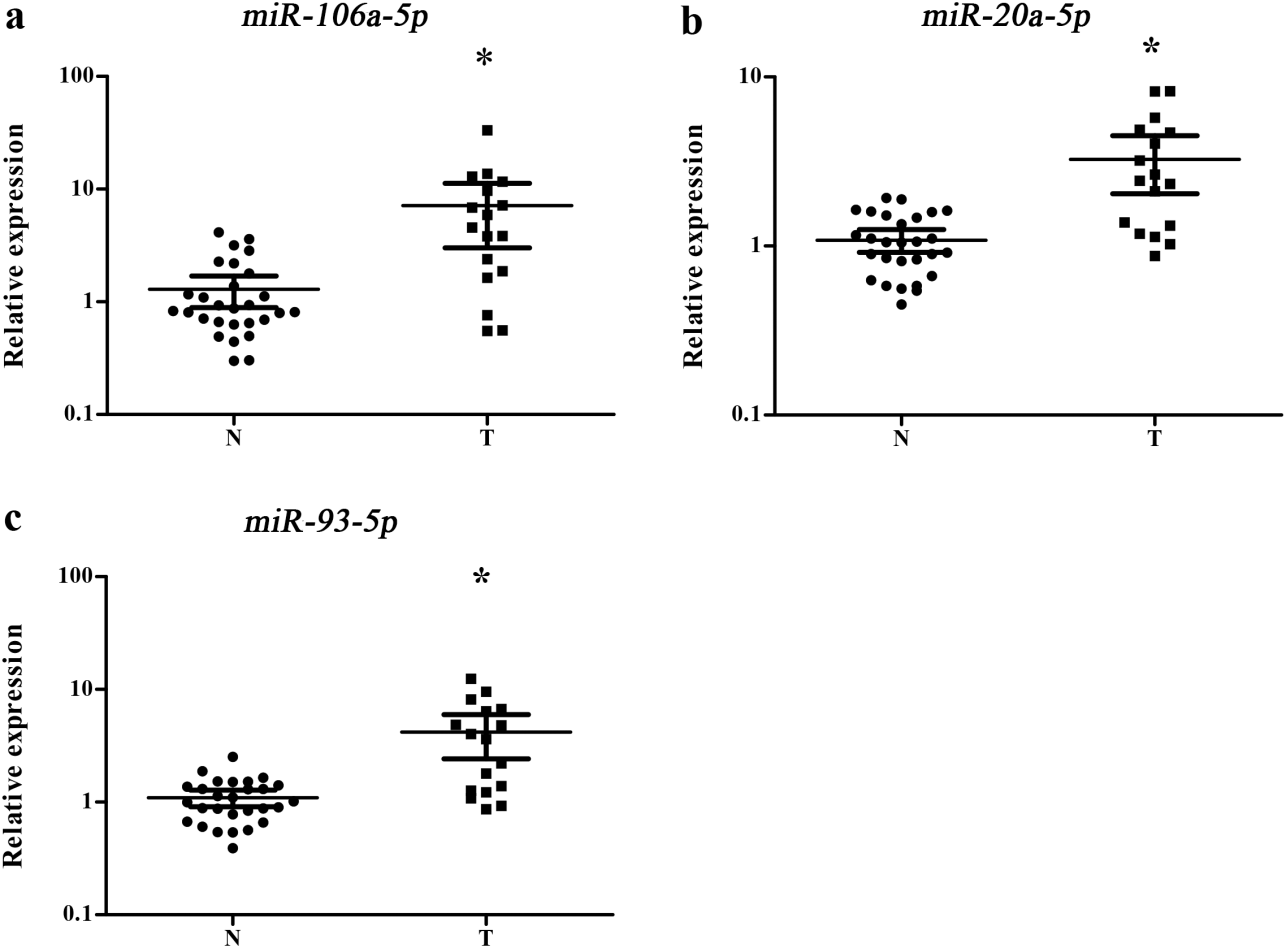
Expression of the three miRNAs in the serum exosomes of 17 lung SCC and 24 NCs N: normal controls; T: tumor. *: P < 0.001.

To further explore the potential form of the three identified miRNAs in serum, esosomal miRNA expression extracted from 17 lung SCC and 28 NC serum samples were examined. As it is shown in Figure 5, the expression of the three miRNAs was all up regulated with statistical significance.

### Comparison of miRNA expression in venous with arterial serum

To discover the difference of miRNA expression between venous and arterial serum, another three pairs of samples were examined. In arterial serum, the expression levels of miR-106a-5p, miR-20a-5p and miR-93-5p were all decreased and yielded borderline statistical significance (*P* = 0.083, 0.066 and 0.071 respectively) (Supplementary Figure 4).

### Bioinformatics analysis of miR-106a-5p, miR-20a-5p and miR-93-5p

The putative target genes of miR-106a-5p, miR-20a-5p and miR-93-5p were identified by DIANA-TarBase v7.0. Then DIANA-miRPath v3.0 was utilized for KEGG pathway analysis (Supplementary Table 4) and GO category analysis (Supplementary Table 5) to investigate the pathways significantly associated with the three miRNAs. All of the three miRNAs are related to pathological mechanisms of cancer such as by interacting with MAPK and mTOR signaling.

## DISCUSSION

In present study, we established a carefully designed procedure to identify a serum miRNA profile of lung SCC in male patients. At the initial screening stage, Exiqon miRNA qPCR panels was utilized to conduct serum miRNA profile, which may be more sensitive and linear in measuring miRNAs with relatively low abundance compared to TaqMan platform. However, the results obtained from pooled samples may be inconsistent with the PCR results conducted on individual serum cases. Hence, our study performed three phases of qRT-PCR validation after the screening phase. In choosing the correct internal reference genes, geNorm was used to assess the most suitable internal reference control. Among the four tested genes, miR-16-5p had the least variation and relatively high levels of expression and was therefore considered as the most reliable gene for normalization. Whether miR-16-5p could be used as a reference molecule in the circulation is still controversial. It has been applied in many previous studies as a reference gene [19], however, it could also be secreted from and influenced by hemolysis [20]. Our result gave a strong support that miR-16-5p is stable in circulation and could be used as a reference gene. Three up-regulated miRNAs (miR-106a-5p, miR-20a-5p and miR-93-5p) were identified and showed high accuracy in the diagnosis of lung SCC (AUC=0.832). The three-miRNA panel also possessed high accuracy in discriminating lung SCC from pulmonary hamartoma (AUC=0.900). In addition, when miR-103a-3p was used for normalization, all of the three miRNAs were statistically significant (Supplementary Figure 5). Therefore, our study hypothesized that these miRNAs might be candidates for the noninvasive lung SCC detection in Chinese male patients.

Previous studies have identified a number of dysregulated serum miRNAs that could distinguish NSCLC patients from NCs. All of the three miRNAs identified in our study have been reported separately to have diagnostic power in NSCLC [21–24]. However, with the increasing understanding of the histologic and molecular differences among different subtypes of NSCLC, sub-grouping NSCLC into different subtypes for individualized treatment is essential both for safety and efficacy outcomes [25]. Recently, altered miRNA expression associated with the diagnosis of lung SCC has been reported by several studies. However, these studies mainly concentrated on tissue samples. The invasive procedures such as surgical section or biopsy to get tissue samples limit its application in the diagnosis of lung SCC. Only one previous study identified a five-miRNA signature (miR-205, miR-19a, miR-19b, miR-20a-5p, miR-451 and miR-30b) in the plasma of lung SCC patients that displayed significantly down-regulation after tumor resection [26]. Among the five miRNAs identified, serum miR-20a-5p was also confirmed in our study that could distinguish lung SCC from NCs. This provided evidence that there was uniformity between serum and plasma in the expression of miRNA. Elevated level of miR-20a-5p could promote growth and inhibit apoptosis in NSCLC cell lines by targeting TβRII [27]. The expression of miR-20a-5p was upregulated in colorectal cancer based on The Cancer Genome Atlas (TCGA) project [28]. Meanwhile, as a member of the miR-17-92 cluster, circulating miR-20a-5p was proved to act as an oncogene in a wide range of other cancer types, such as colorectal cancer [28], gastric cancer [19], esophageal squamous cell carcinoma [29], nasopharyngeal carcinoma [30] as well as astrocytoma [31]. MiR-106a-5p is involved in cell proliferation and metastasis of NSCLC cell lines by targeting *PTEN* [32]. Up-regulation of miR-106a-5p could also enhance the chemoresistance of NSCLC cells to cisplatin by targeting *ABCA1* [33]. Moreover, miR-106a-5p was identified as an angiogenesis-related miRNA [34]. High levels of circulating miR-106a-5p were validated in metastatic breast cancer patients [35], gastric cancer [36] and colorectal cancer [37]. Based on TCGA data set, miR-93-5p was identified to be a member of the pan-cancer oncogenic miRNA superfamily [38]. The overexpression of miR-93-5p could promote tumorigenesis by targeting tumor suppressor *FUS1* [39] and *DAB2* [40]. Meanwhile, it is able to inactivate *ZNRF3* [41], leading to the activation of Wnt signaling, which promote cell proliferation and progression in lung cancer cell lines. Another target gene of miR-93-5p is *LATS2*, the reduced expression of *LATS2* can result in enhanced angiogenesis and metastasis [42]. Elevated level of circulating miR-93-5p was identified as a diagnosis biomarker in ovarian cancer [43] and colorectal cancer [44].

The origin and secretory mechanism of circulating miRNAs remains a hot concern. Some studies believed that circulating miRNAs were generated from primary tumor tissues and then released into the blood [45, 46]. Thus, we verified the expression of the three miRNAs (miR-106a-5p, miR-20a-5p and miR-93-5p) in lung SCC tissues. All of the three miRNAs were up-regulated in tissue samples, which is in line with the theory. The mechanism behind the stability of circulating miRNAs is still unclear. Recent studies have revealed that miRNAs may be protected by forming complexes with proteins such as Argonaute and GW182 or in microvesicles or small membrane vesicles of endocytic origin called exosomes. Exosomes are 40–100 nm nano-sized vesicles that contain biologically active proteins and miRNAs [47]. Tumor-derived exosmoes are emerging as important mediators of tumorigenesis and immune escape [48]. So, our study further explored exosomal serum miRNAs to identify the potential form of the three miRNAs in the extracellular environment. The expression of miR-106a-5p, miR-20a-5p and miR-93-5p was all up-regulated in serum exosomes of lung SCC patients. Previously, the overexpression of serum exosomal miR-106a-5p and miR-20a-5p was reported to enhance cell proliferation and differentiation by down-regulating the MARK1 signaling pathway in nasopharyngeal carcinoma (NPC) [48]. The three identified miRNAs demonstrated a relatively abundant concentration in venous serum than that in arterial serum in our study. The phenomenon may be partly explained that hypoxia could increase the expression of the three miRNAs and may promote angiogenesis. Further studies are needed to find the exact mechanism.

Although the results are promising, a major limitation of our study was unable to detect the dynamic changes of serum miRNAs before and after surgery. Another limitation is that the number of patients enrolled was relatively small. Prospective studies on larger cohorts of patients are needed to confirm the diagnostic value of these miRNAs. In conclusion, our study identified a three-miRNA panel (miR-106a-5p, miR-20a-5p and miR-93-5p) in the serum of male lung SCC patients that could serve as an effective non-invasive biomarker for the diagnosis of lung SCC. Further studies involving larger cohorts are needed to validate the clinical application of these serum miRNAs.

## MATERIALS AND METHODS

### Study design and study population

Our study enrolled 102 patients who underwent lung tumor resection and 10 patients with pulmonary hamartoma at Jiangshu Cancer Hospital between 2012 and 2015. All the patients were male and histopathologically confirmed as lung SCC. 108 male normal controls (NCs) from the First Affiliated Hospital of Nanjing Medical University were acquired. The peripheral blood was drawn before surgery and none of the patients had received preoperative radiation or chemotherapy. Clinical and histopathological features of patients including age, smoking, TNM stage (according to the seventh edition American Joint Committee on Cancer (AJCC), differentiation degree were obtained retrospectively from the patients’ records. Our study was approved by the institutional review board of Nanjing Medical University and the Hospital Ethics Committee. Written informed consent was obtained from every participant.

A multiphase study was designed to identify specific serum miRNAs for the diagnosis of lung SCC (Figure 1). In the initial screening stage, 30 serum samples from lung SCC patients and 10 from NCs were randomly selected and pooled as 3 lung SCC samples and 1 NC sample (10 samples were pooled as 1 sample). ExiqonmiRCURY-Ready-to-Use PCR-Human-panel-I+II-V1.M (Exiqon miRNA qPCR panel, Vedbaek, Denmark) were applied to identify miRNAs whose expression were altered in lung SCC samples compared to NCs as previously described. In the training stage, serum samples from 24 lung SCC patients and 15 NCs were used to confirm the dysregulated miRNAs assessed by the screening stage. Subsequently in the testing stage, we refined the miRNAs by 44 lung SCC patients and 57 NCs. An external patient cohort of 34 lung SCC patients and 36 NCs were used to assess the diagnostic value of the selected miRNAs. Additionally, another 10 patients with pulmonary hamartoma was used to validate the diagnostic performance of the candidate miRNAs. The panel of serum miRNAs was further validated in 23 pairs of tissue specimens and matched normal tissues. Serum exosomal miRNAs were also identified in 17 lung SCC patients and 28 NCs to further discover the potential form of miRNAs in the peripheral blood. Arterial and venous blood samples from 3 lung SCC patients were obtained to compare the difference of miRNA expression between peripheral arterial and venous serum.

Venous blood samples of healthy controls and lung SCC patients without initial treatment were collected and placed in a serum separator tube. Cell-free serum was isolated from blood samples within 12 hours after collection using a two-step protocol (1,500 r.p.m. for 10 min, 12,000 r.p.m. for 2 min) to completely remove cell debris and then stored at − 80 °C until analysis. Tissue specimens were collected from surgery patients and kept in liquid nitrogen.

### Isolation of exosomes

Exosomes were isolated from serum using ExoQuick™ (System Biosciences, Mountain View, Calif) according to the manufacturer’s protocol. Precipitated from 50 μ l ExoQuickexosome precipitation solutions and 200 μ l serum, exosome pellets were lysed in 200 μ l RNase-free water for further processing.

### RNA extraction

Total RNA was extracted from 200μl serum or exosome using the mirVana PARIS Kit (Ambion, Austin, TX, USA) according to the manufacturer’s protocol. 5μl of synthetic C.elegans *miR-39* (5 nM/L, RiboBio, Guangzhou, China) was added to each sample after the addition of denaturing solution (Ambion, Austin, TX, USA) for normalization. Trizol (Invitrogen, Carlsbad, CA, USA) was used to extract total RNA from tissue samples. Finally, total RNA was dissolved in 100μl RNase-free water and kept at -80°C until further analysis. The ultraviolet spectrophotometer was applied to evaluate the concentration and purity of the extracted total RNA.

### Quantitative real-time polymerase chain reaction (qRT-PCR)

The specific primers of reverse transcption (RT) and polymerase chain reaction (PCR) (RiboBio, China) were used to amplify miRNAs. The process of RT and PCR were performed as described previously [49–51]. MiRNAs were amplified and detected in a LightCycler 480 (Roche 480, Germany) real-time thermal cycler, using SYBR Green dye. The expression of miRNAs in serum and exosomes was calculated using the 2^−ΔΔCt^ method relative to the combination of exogenous reference miRNA (*cel-miR-39*) and endogenous reference miRNA (miR-16), ΔCt = CtmiRNA− 1/2 (Ct*cel-miR-39* + CtmiR-16) [52]. The relative expression level of tissue miRNAs were calculated using the 2^−ΔΔCt^ method and normalized to that of *RNU6B (U6).*

### Statistical analysis

GeNorm Version 3.5 was used to assess the expression stability of serum miRNA and the stability value (M) was calculated. Mann-Whitney test was used to analyze differential miRNAs expression between lung SCC patients and NCs. The association between miRNAs and the clinical characteristics was evaluated by χ2 test or paired-samples t test. Receiver operating characteristic (ROC) curves and the area under the ROC curve (AUC) were used to estimate the diagnostic value of the candidate miRNAs for lung SCC. All the statistical analyses were performed using SPSS software (version 20.0, IBM, USA). A two-sided *P* value <0.05 was considered statistically significant.

## SUPPLEMENTARY MATERIALS FIGURES AND TABLES





## Abbreviations

miRNA: microRNA
NSCLC: Non-small cell lung cancer
SCC: squamous cell carcinoma
TCGA: The Cancer Genome Atlas
qRT-PCR: quantitative real-time polymerase chain reaction
NC: normal control
ROC: receiver operating characteristic
LDCT: Low-dose computed tomography; survival
SCC Ag: squamous cell carcinoma antigen
NSE: neuron-specificenolase
AJCC: American Joint Committee on Cancer
