# Supplementary material for: A three-microRNA signature for lung squamous cell carcinoma diagnosis in Chinese male patients
Source: Oncotarget. 2017 Jul 28;8(49):86897–907. doi: 10.18632/oncotarget.19666 (PMC5689734; doi:10.18632/oncotarget.19666)
Supplement: Supplementary file 1 [file oncotarget-08-86897-s001.pdf]

A three-microRNA signature for lung squamous cell carcinoma diagnosis in Chinese male patients

SUPPLEMENTARY MATERIALS

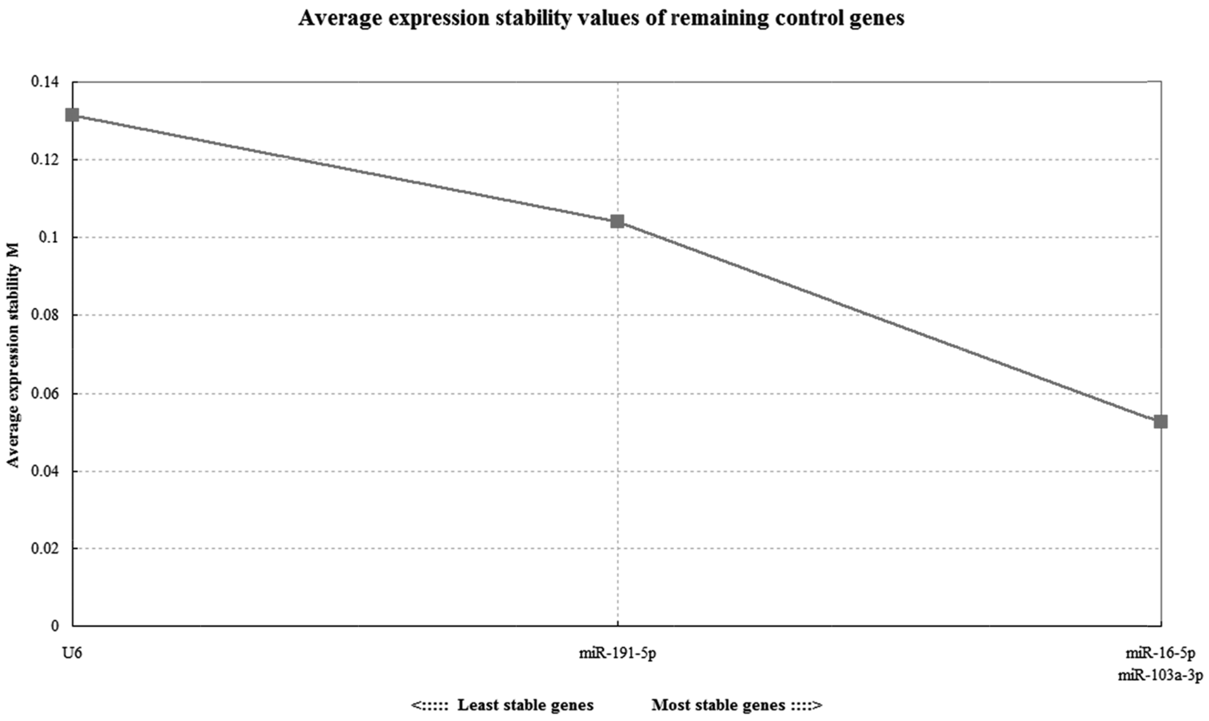

Supplementary Figure 1: GeNorm analysis of candidate reference genes. Ranking of candidate reference genes according to average expression stability. The least stable gene with the highest stability measure. M was excluded in a stepwise manner until two most stable genes remained: miR-16-5p and miR-103a-3p.

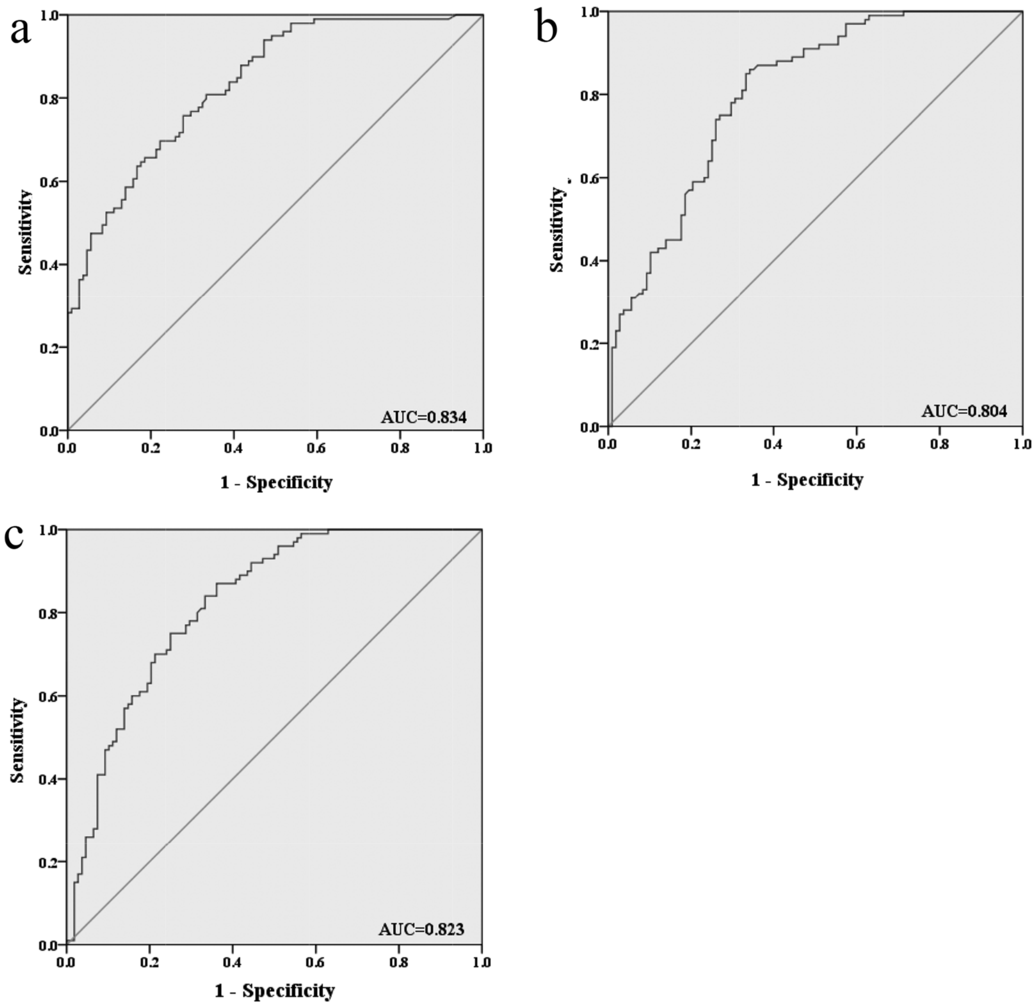

Supplementary Figure 2: ROC curve analyses of each miRNA to discriminate lung SCC patients from NCs in the combined three phases (102 SCC VS. 108 NCs). (a) miR-106a-5p; **(b)** miR-20a-5p; **(c)** miR-93-5p.

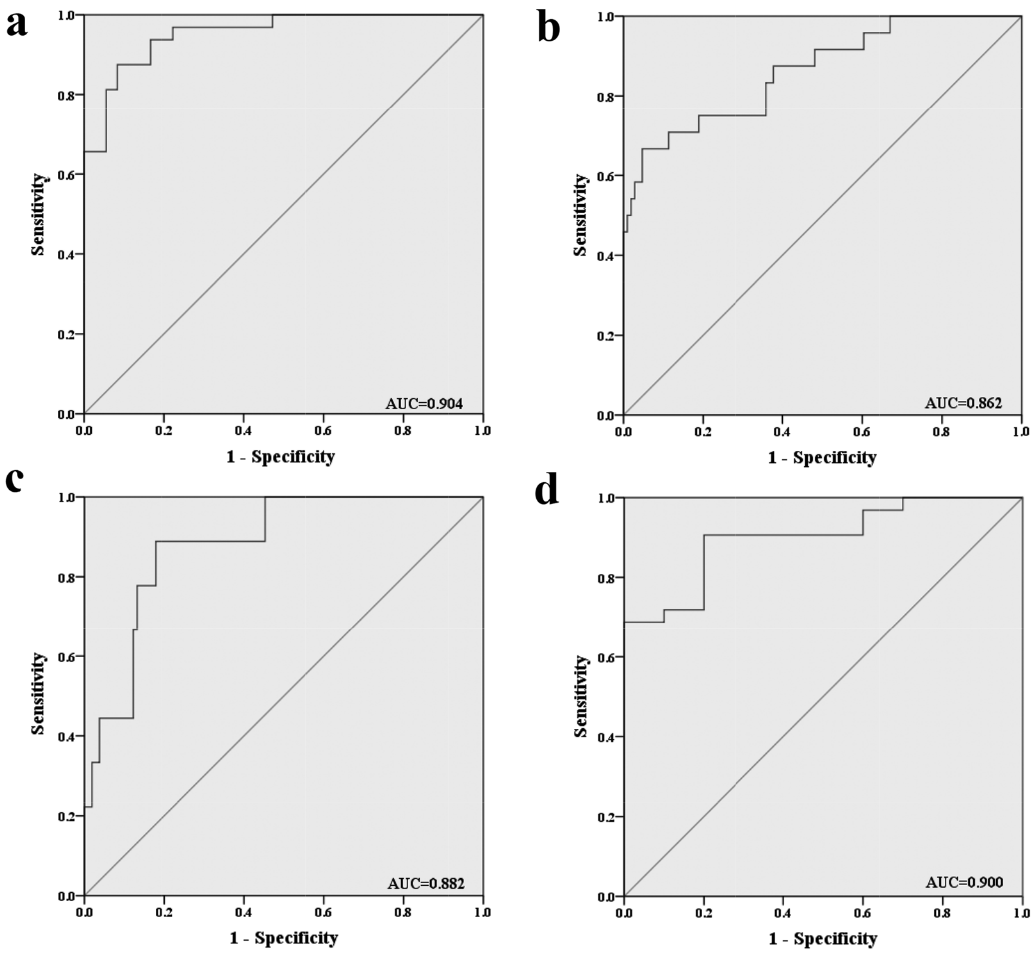

Supplementary Figure 3: ROC curves for the ability of the three-miRNA panel to differentiate lung SCC patients with different TNM stages and from pulmonary hamartoma. **(a)** stages I(40 SCC VS. 40 NCs), **(b)** stage II(46 SCC VS. 52 NCs), **(c)** stage III(16 SCC VS. 16 NCs), **(b)** stage II, **(c)** stage III, **(d)** 32 SCC VS. 10 pulmonary hamartoma.

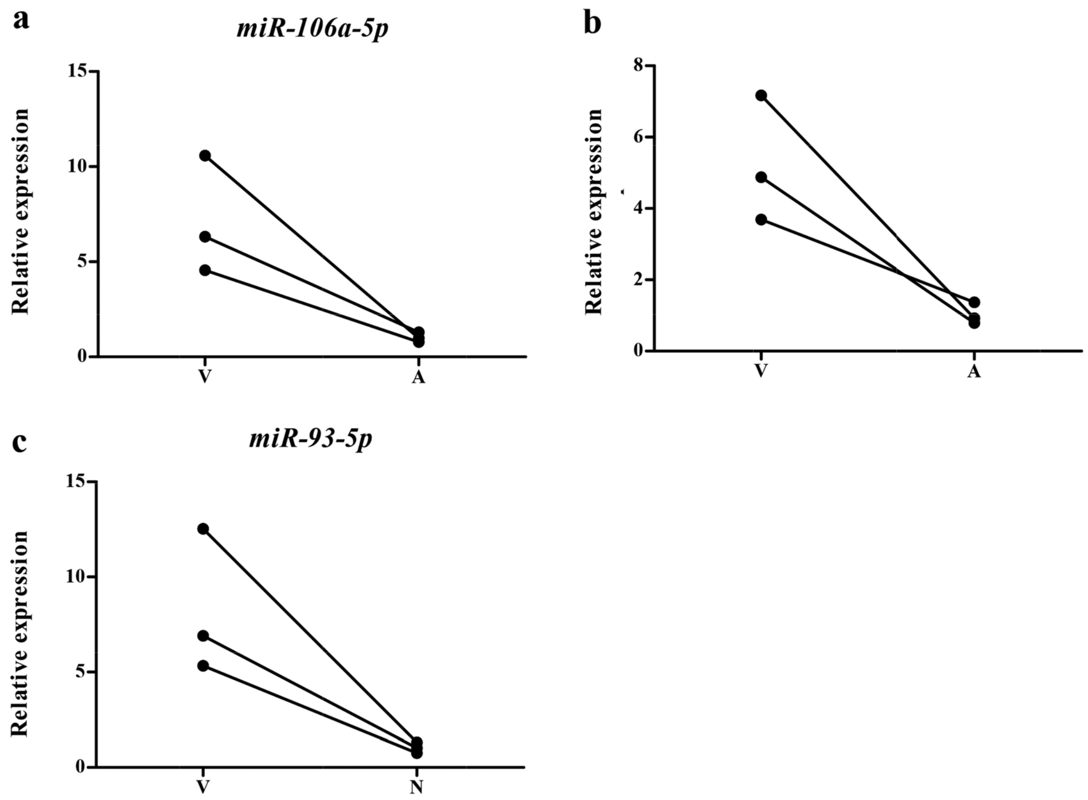

Supplementary Figure 4: Expression levels of the three miRNAs in the venous and arterial serum of three pairs of samples. (a) miR-106a-5p; **(b)** miR-20a-5p; **(c)** miR-93-5p; V: venous serum; A: arterial serum. *P* value was 0.083, 0.066 and 0.071 respectively.

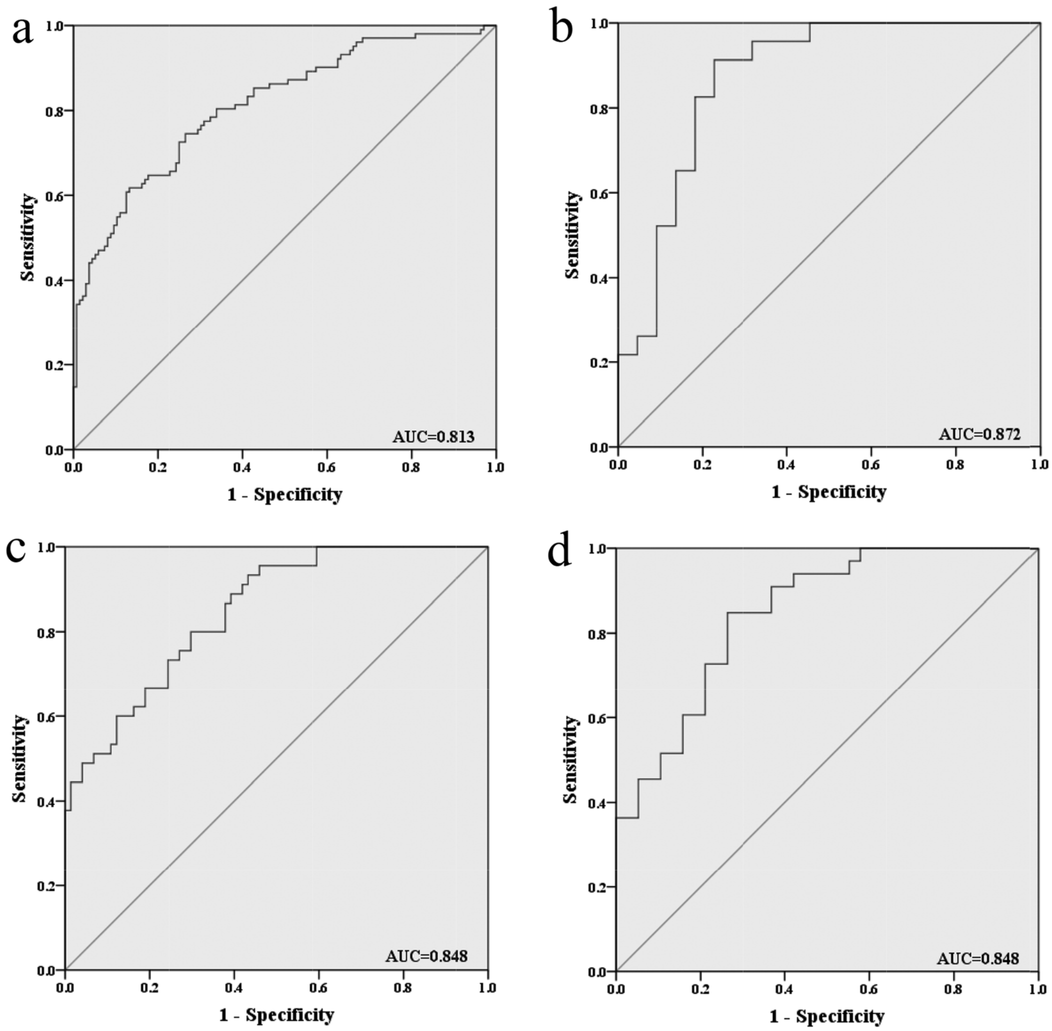

Supplementary Figure 5: ROC curves for the three - miRNA panel to discriminate lung SCC patients from NCs (miR-103a-3p as an internal reference control). **(a)** the combined three phases of training, testing and external validation phases (102 SCC VS. 108 NCs); **(b)** training phase (24 SCC VS. 15 NCs); **(c)** testing phase (44 SCC VS. 57 NCs). **(d)** external validation (34 SCC VS. 36 NCs).

Supplementary Table 1: Differently expressed miRNAs in the screening phase

MiRNA	Fold change			Mean fold
	Pool 1	Pool 2	Pool 3	
miR-101-3p	1.98	2.41	1.62	2.00
**miR-106a-5p**	10.01	8.23	8.09	8.78
miR-106b-3p	1.91	2.00	1.99	1.97
miR-133b	1.79	2.79	2.87	2.48
miR-136-5p	1.69	1.71	2.57	1.99
**miR-144-3p**	3.56	2.87	2.98	3.14
**miR-155-5p**	3.08	2.20	2.12	2.47
**miR-15b-5p**	4.10	3.78	3.99	3.96
miR-16-2-3p	2.89	3.76	2.51	3.05
miR-185-5p	6.08	5.90	4.65	5.54
miR-18b-5p	3.56	2.00	2.78	2.78
**miR-195-5p**	2.41	6.13	2.89	3.81
miR-205-5p	2.26	3.34	2.16	2.59
**miR-20a-3p**	5.20	5.99	5.07	5.42
**miR-20a-5p**	5.46	3.05	5.24	4.58
miR-23a-3p	3.52	2.44	2.17	2.71
miR-28-5p	3.35	3.61	9.91	5.62
miR-29a-5p	3.57	3.11	3.47	3.38
miR-29b-3p	1.89	2.21	1.97	2.02
miR-301a-3p	3.41	3.95	3.21	3.52
miR-32-5p	2.81	5.69	3.68	4.06
miR-335-5p	1.99	2.96	1.02	1.99
miR-424-5p	5.27	4.68	6.29	5.41
**miR-451a**	3.14	2.96	2.88	2.99
**miR-93-5p**	5.92	4.83	7.04	5.93
**miR-122-5p**	-1.95	-2.51	-2.47	-2.31
**miR-150-5p**	-2.02	-2.18	-2.15	-2.12
miR-190a	-2.58	-3.04	-2.36	-2.66
miR-193b-3p	-2.34	-2.09	-1.52	-1.98
**miR-199a-5p**	-2.92	-1.70	-1.55	-2.06
miR-19a-3p	-1.62	-2.22	-2.71	-2.18
miR-200a-3p	-2.58	-2.99	-19.8	-8.46
**miR-326**	-2.00	-1.98	-2.05	-2.01
miR-328	-1.61	-1.50	-1.85	-1.65
miR-485-3p	-4.70	-12.3	-19.9	-12.3
miR-543	-11.8	-17.1	-19.6	-16.2
**miR-574-3p**	-2.74	-2.62	-2.33	-2.56
miR-766-3p	-4.64	-2.92	-2.61	-3.39

Note: miRNAs in bold were identified after the training phase.

Supplementary Table 2: Expression levels of the four reference genes in the peripheral serum (presented as mean ± SD)

Name	Expression
MiR-16-5p	25.3 ±2.10
MiR-103a-5p	32.1 ±1.27
MiR-191-5p	30.8 ±3.62
U6	20.3 ±2.68

Supplementary Table 3: Expression levels of the three miRNAs in the peripheral serum in the training and testing stages (presented as mean ± SD). FC: fold change

miRNA	Training stage				Testing stage				Combined	
	Controls	Cases	FC	*P* value	Controls	Cases	FC	*P* value	FC	*P* value
**miR-106a-5p**	3.49±2.90	7.94±5.16	2.27	0.004	1.76±2.10	15.0±18.2	8.52	<0.001	5.88	<0.001
**miR-20a-5p**	1.13±0.65	4.59±3.37	4.05	<0.001	1.32±1.31	4.24±4.16	3.22	<0.001	3.42	<0.001
**miR-93-5p**	1.23±0.99	4.04±2.63	3.28	<0.001	1.31±1.73	3.36±2.56	2.57	<0.001	2.79	<0.001

Supplementary Table 4: KEGG pathway analysis for miR-106a-5p, miR-20a-5p and miR-93-5p

See Supplementary File 1

Supplementary Table 5: GO category analysis for miR-106a-5p, miR-20a-5p and miR-93-5p

See Supplementary File 1
