# Supplementary material for: A three-microRNA signature for lung squamous cell carcinoma diagnosis in Chinese male patients
Source: Oncotarget. 2017 Jul 28;8(49):86897–907. doi: 10.18632/oncotarget.19666 (PMC5689734; doi:10.18632/oncotarget.19666)
Supplement: Supplementary file 2 [file oncotarget-08-86897-s002.docx]

**Table S4. KEGG pathway analysis for miR-106a-5p, miR-20a-5p and miR-93-5p**

| **miR-106a-5p** | ***P value*** | **miR-20a-5p** | ***P value*** | **miR-93-5p** | ***P value*** |
| --- | --- | --- | --- | --- | --- |
| Proteoglycans in cancer | 1.02E-06 | Protein processing in endoplasmic reticulum | 1.06E-05 | Prion diseases | 5.15E-17 |
| Hippo signaling pathway | 4.29E-06 | Cell cycle | 2.06E-05 | Fatty acid biosynthesis | 5.58E-16 |
| ECM-receptor interaction | 4.29E-06 | Pathways in cancer | 2.06E-05 | mRNA surveillance pathway | 1.37E-06 |
| FoxO signaling pathway | 7.81E-05 | Circadian rhythm | 8.20E-05 | Viral carcinogenesis | 2.20E-05 |
| TGF-beta signaling pathway | 7.81E-05 | Hepatitis B | 8.20E-05 | FoxO signaling pathway | 6.96E-05 |
| Focal adhesion | 0.0001505 | mRNA surveillance pathway | 0.0001471 | TGF-beta signaling pathway | 0.0001191 |
| Pathways in cancer | 0.0001505 | Chronic myeloid leukemia | 0.0001747 | Hepatitis B | 0.0001191 |
| Renal cell carcinoma | 0.0005371 | Proteoglycans in cancer | 0.0002661 | Proteoglycans in cancer | 0.0001191 |
| Hepatitis C | 0.0019923 | Bladder cancer | 0.0003574 | Glioma | 0.0001191 |
| Colorectal cancer | 0.0019923 | FoxO signaling pathway | 0.0004075 | Chronic myeloid leukemia | 0.0001421 |
| Lysine degradation | 0.0024921 | TGF-beta signaling pathway | 0.0005351 | Colorectal cancer | 0.0001847 |
| Endocytosis | 0.0044436 | Renal cell carcinoma | 0.0010342 | Pathways in cancer | 0.0001944 |
| Protein processing in endoplasmic reticulum | 0.0049613 | Glioma | 0.0010342 | Bladder cancer | 0.0003179 |
| Chronic myeloid leukemia | 0.0049613 | Prostate cancer | 0.0010342 | Prolactin signaling pathway | 0.000345 |
| Steroid biosynthesis | 0.0057219 | MAPK signaling pathway | 0.0011049 | Hippo signaling pathway | 0.0004071 |
| Thyroid cancer | 0.0067376 | N-Glycan biosynthesis | 0.001243 | Pancreatic cancer | 0.0004578 |
| Glioma | 0.0071833 | mTOR signaling pathway | 0.0014341 | Signaling pathways regulating pluripotency of stem cells | 0.0006013 |
| Adherens junction | 0.0112487 | Sphingolipid signaling pathway | 0.0019841 | MAPK signaling pathway | 0.0006272 |
| Pancreatic cancer | 0.0143059 | Colorectal cancer | 0.0019841 | RNA transport | 0.0007938 |
| Prostate cancer | 0.0144707 | Focal adhesion | 0.002278 | Ubiquitin mediated proteolysis | 0.0007938 |
| MAPK signaling pathway | 0.0148921 | Glycosaminoglycan biosynthesis - keratan sulfate | 0.0040943 | Renal cell carcinoma | 0.0007938 |
| Non-small cell lung cancer | 0.0200259 | p53 signaling pathway | 0.0042128 | Melanoma | 0.0007938 |
| Circadian rhythm | 0.0201942 | Hippo signaling pathway | 0.0054195 | Sphingolipid signaling pathway | 0.0009903 |
| Hepatitis B | 0.0217019 | Hepatitis C | 0.0057706 | p53 signaling pathway | 0.0011217 |
| Acute myeloid leukemia | 0.0217019 | Regulation of actin cytoskeleton | 0.0059457 | Cell cycle | 0.0012456 |
| Ubiquitin mediated proteolysis | 0.0229041 | Melanoma | 0.0059457 | Oocyte meiosis | 0.0012499 |
| Endometrial cancer | 0.0229041 | Pancreatic cancer | 0.0059457 | Lysine degradation | 0.0014882 |
| Prolactin signaling pathway | 0.0278992 | Insulin signaling pathway | 0.0068793 | Prostate cancer | 0.0016703 |
| Wnt signaling pathway | 0.0297859 | Thyroid cancer | 0.0068793 | Insulin signaling pathway | 0.0016711 |
| Gap junction | 0.0297859 | Wnt signaling pathway | 0.0078906 | mTOR signaling pathway | 0.0016808 |
| Signaling pathways regulating pluripotency of stem cells | 0.034027 | Adherens junction | 0.0080723 | Thyroid cancer | 0.0017826 |
| Axon guidance | 0.0430211 | RNA transport | 0.0088785 | Neurotrophin signaling pathway | 0.0024989 |
|  |  | Acute myeloid leukemia | 0.0088785 | Progesterone-mediated oocyte maturation | 0.0024989 |
|  |  | Small cell lung cancer | 0.0088785 | Bacterial invasion of epithelial cells | 0.0024989 |
|  |  | Oocyte meiosis | 0.0089726 | Fatty acid metabolism | 0.0029861 |
|  |  | Viral carcinogenesis | 0.0089726 | Endometrial cancer | 0.0033907 |
|  |  | Lysine degradation | 0.0096957 | Hepatitis C | 0.0041643 |
|  |  | Steroid biosynthesis | 0.0146653 | Steroid biosynthesis | 0.0041668 |
|  |  | Axon guidance | 0.0160098 | RNA degradation | 0.0058821 |
|  |  | PI3K-Akt signaling pathway | 0.0177686 | Non-small cell lung cancer | 0.0067932 |
|  |  | Non-small cell lung cancer | 0.0232245 | Protein processing in endoplasmic reticulum | 0.0118834 |
|  |  | RNA degradation | 0.0302135 | Thyroid hormone signaling pathway | 0.015776 |
|  |  | Ubiquitin mediated proteolysis | 0.0302135 | AMPK signaling pathway | 0.0163921 |
|  |  | Endocytosis | 0.0302135 | Wnt signaling pathway | 0.0163921 |
|  |  | DNA replication | 0.0351982 | Transcriptional misregulation in cancer | 0.0163921 |
|  |  | Neurotrophin signaling pathway | 0.0389645 | HTLV-I infection | 0.021032 |
|  |  | Long-term depression | 0.0389645 | Shigellosis | 0.0243861 |
|  |  | Thyroid hormone signaling pathway | 0.0389645 | PI3K-Akt signaling pathway | 0.0261421 |
|  |  | Adrenergic signaling in cardiomyocytes | 0.0488359 | Acute myeloid leukemia | 0.0275523 |
|  |  | Signaling pathways regulating pluripotency of stem cells | 0.0488359 | Focal adhesion | 0.0278225 |
|  |  | Progesterone-mediated oocyte maturation | 0.0488359 | Choline metabolism in cancer | 0.0278225 |
|  |  | Glycosaminoglycan biosynthesis - chondroitin sulfate / dermatan sulfate | 0.0492529 | Regulation of actin cytoskeleton | 0.0293748 |

**Table S5. GO category analysis for miR-106a-5p, miR-20a-5p and miR-93-5p.**

| **miR-106a-5p** | ***P value*** | **miR-20a-5p** | ***P value*** | **miR-93-5p** | ***P value*** |
| --- | --- | --- | --- | --- | --- |
| organelle | 5.29E-96 | organelle | 3.93E-165 | organelle | 2.62E-176 |
| ion binding | 1.38E-42 | cellular nitrogen compound metabolic process | 1.99E-76 | cellular nitrogen compound metabolic process | 8.24E-87 |
| cellular nitrogen compound metabolic process | 1.20E-38 | cellular protein modification process | 2.57E-56 | cellular protein modification process | 3.97E-60 |
| cellular protein modification process | 1.70E-38 | ion binding | 1.20E-48 | ion binding | 4.07E-56 |
| enzyme binding | 4.64E-25 | gene expression | 5.19E-46 | biosynthetic process | 1.31E-54 |
| molecular_function | 2.88E-23 | biosynthetic process | 9.80E-46 | gene expression | 1.65E-47 |
| biosynthetic process | 6.12E-22 | molecular_function | 1.73E-40 | enzyme binding | 7.14E-42 |
| cytosol | 3.65E-19 | enzyme binding | 1.19E-35 | molecular_function | 4.27E-39 |
| response to stress | 2.01E-18 | nucleoplasm | 5.27E-34 | protein complex | 9.28E-39 |
| biological_process | 8.02E-18 | mitotic cell cycle | 1.36E-32 | nucleoplasm | 7.41E-35 |
| cellular_component | 8.02E-18 | protein complex | 3.30E-31 | cytosol | 5.03E-32 |
| gene expression | 9.39E-17 | cellular_component | 5.60E-31 | viral process | 1.06E-30 |
| symbiosis, encompassing mutualism through parasitism | 1.05E-16 | cytosol | 5.55E-29 | symbiosis, encompassing mutualism through parasitism | 1.31E-29 |
| viral process | 1.88E-15 | catabolic process | 4.56E-26 | catabolic process | 2.74E-29 |
| cell death | 3.58E-15 | biological_process | 3.26E-25 | cellular_component | 5.02E-29 |
| catabolic process | 9.84E-15 | symbiosis, encompassing mutualism through parasitism | 2.62E-23 | mitotic cell cycle | 4.99E-25 |
| cellular component assembly | 1.75E-14 | viral process | 1.20E-22 | RNA binding | 2.18E-24 |
| protein complex | 1.75E-14 | neurotrophin TRK receptor signaling pathway | 2.78E-21 | neurotrophin TRK receptor signaling pathway | 1.11E-22 |
| nucleoplasm | 2.60E-13 | response to stress | 3.14E-21 | biological_process | 1.11E-22 |
| neurotrophin TRK receptor signaling pathway | 2.91E-11 | protein binding transcription factor activity | 6.96E-20 | nucleobase-containing compound catabolic process | 9.75E-22 |
| enzyme regulator activity | 3.48E-11 | nucleic acid binding transcription factor activity | 7.51E-19 | response to stress | 9.84E-18 |
| small molecule metabolic process | 4.20E-11 | RNA binding | 3.58E-16 | protein binding transcription factor activity | 4.80E-17 |
| macromolecular complex assembly | 6.65E-11 | nucleobase-containing compound catabolic process | 2.57E-15 | Fc-epsilon receptor signaling pathway | 2.01E-16 |
| protein binding transcription factor activity | 1.55E-10 | Fc-epsilon receptor signaling pathway | 1.44E-14 | cellular protein metabolic process | 2.02E-15 |
| RNA binding | 1.55E-10 | cell death | 2.05E-13 | nucleic acid binding transcription factor activity | 2.97E-15 |
| nucleic acid binding transcription factor activity | 4.74E-10 | cellular protein metabolic process | 7.04E-12 | mRNA metabolic process | 2.98E-15 |
| nucleobase-containing compound catabolic process | 6.66E-10 | G2/M transition of mitotic cell cycle | 2.18E-11 | DNA metabolic process | 3.09E-15 |
| activation of signaling protein activity involved in unfolded protein response | 1.56E-09 | small molecule metabolic process | 2.18E-11 | membrane organization | 1.01E-14 |
| mitotic cell cycle | 6.64E-09 | cellular component assembly | 2.71E-11 | transcription, DNA-templated | 6.18E-14 |
| membrane organization | 3.23E-08 | membrane organization | 9.12E-11 | small molecule metabolic process | 5.68E-13 |
| cell cycle arrest | 1.55E-07 | blood coagulation | 1.38E-10 | cell death | 2.23E-12 |
| protein complex assembly | 1.55E-07 | macromolecular complex assembly | 4.22E-10 | cellular component assembly | 2.52E-12 |
| cellular protein metabolic process | 6.05E-07 | mRNA metabolic process | 4.37E-10 | poly(A) RNA binding | 5.48E-12 |
| small conjugating protein binding | 6.48E-07 | DNA metabolic process | 4.37E-10 | macromolecular complex assembly | 7.46E-12 |
| microtubule organizing center | 6.48E-07 | transcription, DNA-templated | 6.68E-10 | RNA metabolic process | 1.00E-11 |
| Fc-epsilon receptor signaling pathway | 2.04E-06 | cell cycle | 2.48E-09 | blood coagulation | 1.33E-11 |
| G2/M transition of mitotic cell cycle | 2.04E-06 | microtubule organizing center | 6.70E-09 | fibroblast growth factor receptor signaling pathway | 4.48E-11 |
| immune system process | 1.95E-05 | epidermal growth factor receptor signaling pathway | 1.91E-08 | cytoskeletal protein binding | 1.88E-10 |
| endoplasmic reticulum unfolded protein response | 2.87E-05 | RNA metabolic process | 1.91E-08 | enzyme regulator activity | 1.15E-09 |
| cell junction organization | 4.75E-05 | enzyme regulator activity | 2.43E-08 | epidermal growth factor receptor signaling pathway | 1.36E-09 |
| cytoskeletal protein binding | 5.32E-05 | Fc-gamma receptor signaling pathway involved in phagocytosis | 3.37E-08 | nucleocytoplasmic transport | 6.99E-09 |
| poly(A) RNA binding | 0.0001506 | protein complex assembly | 9.82E-08 | small conjugating protein binding | 5.31E-08 |
| regulation of transcription from RNA polymerase II promoter in response to hypoxia | 0.0001658 | cytoskeletal protein binding | 1.97E-07 | insulin receptor signaling pathway | 9.27E-08 |
| Ras protein signal transduction | 0.0001724 | mRNA processing | 9.64E-07 | phosphatidylinositol-mediated signaling | 9.40E-08 |
| mRNA metabolic process | 0.0001863 | fibroblast growth factor receptor signaling pathway | 1.33E-06 | protein complex assembly | 1.15E-07 |
| nucleocytoplasmic transport | 0.0002376 | activation of signaling protein activity involved in unfolded protein response | 1.35E-06 | G1/S transition of mitotic cell cycle | 4.15E-07 |
| negative regulation of cell proliferation | 0.0004579 | G1/S transition of mitotic cell cycle | 1.37E-06 | transforming growth factor beta receptor signaling pathway | 2.23E-06 |
| fibroblast growth factor receptor signaling pathway | 0.000463 | positive regulation of protein insertion into mitochondrial membrane involved in apoptotic signaling pathway | 1.76E-06 | cell cycle | 2.23E-06 |
| blood coagulation | 0.0004859 | immune system process | 1.95E-06 | activation of signaling protein activity involved in unfolded protein response | 2.36E-06 |
| Fc-gamma receptor signaling pathway involved in phagocytosis | 0.0005527 | transcription initiation from RNA polymerase II promoter | 2.09E-06 | positive regulation of protein insertion into mitochondrial membrane involved in apoptotic signaling pathway | 2.68E-06 |
| toll-like receptor 10 signaling pathway | 0.0008063 | small conjugating protein binding | 2.16E-06 | Fc-gamma receptor signaling pathway involved in phagocytosis | 2.74E-06 |
| toll-like receptor 9 signaling pathway | 0.0009208 | poly(A) RNA binding | 2.59E-06 | hexose transport | 2.83E-06 |
| stress-activated MAPK cascade | 0.0011187 | innate immune response | 3.73E-06 | chromatin organization | 5.20E-06 |
| toll-like receptor TLR1:TLR2 signaling pathway | 0.0013611 | termination of RNA polymerase II transcription | 4.96E-06 | protein polyubiquitination | 5.20E-06 |
| toll-like receptor TLR6:TLR2 signaling pathway | 0.0013611 | intrinsic apoptotic signaling pathway | 1.55E-05 | mRNA processing | 5.20E-06 |
| DNA metabolic process | 0.0020142 | apoptotic signaling pathway | 1.55E-05 | transcription initiation from RNA polymerase II promoter | 5.87E-06 |
| JAK-STAT cascade involved in growth hormone signaling pathway | 0.0020694 | transforming growth factor beta receptor signaling pathway | 1.57E-05 | mitotic nuclear envelope disassembly | 6.08E-06 |
| toll-like receptor 5 signaling pathway | 0.0022345 | phosphatidylinositol-mediated signaling | 2.37E-05 | cell cycle arrest | 8.22E-06 |
| kinase activity | 0.0024508 | platelet activation | 4.07E-05 | microtubule organizing center | 8.39E-06 |
| toll-like receptor signaling pathway | 0.0024709 | nucleocytoplasmic transport | 4.07E-05 | G2/M transition of mitotic cell cycle | 1.05E-05 |
| TRIF-dependent toll-like receptor signaling pathway | 0.002571 | post-translational protein modification | 5.14E-05 | regulation of ubiquitin-protein ligase activity involved in mitotic cell cycle | 1.78E-05 |
| transcription, DNA-templated | 0.002571 | cell cycle arrest | 5.92E-05 | cellular lipid metabolic process | 2.19E-05 |
| cytoskeleton organization | 0.0026729 | transcription from RNA polymerase II promoter | 6.26E-05 | intrinsic apoptotic signaling pathway | 2.57E-05 |
| virion assembly | 0.0027072 | mRNA 3'-end processing | 6.96E-05 | regulation of glucose transport | 3.08E-05 |
| RNA metabolic process | 0.0030522 | RNA splicing | 6.96E-05 | nuclear-transcribed mRNA catabolic process, deadenylation-dependent decay | 4.23E-05 |
| cellular component disassembly involved in execution phase of apoptosis | 0.0040238 | insulin receptor signaling pathway | 8.37E-05 | termination of RNA polymerase II transcription | 5.06E-05 |
| phosphatidylinositol-mediated signaling | 0.0043709 | nuclear-transcribed mRNA catabolic process, deadenylation-dependent decay | 0.0001286 | toll-like receptor 10 signaling pathway | 5.06E-05 |
| epidermal growth factor receptor signaling pathway | 0.0043709 | nuclear-transcribed mRNA poly(A) tail shortening | 0.0002161 | positive regulation of ubiquitin-protein ligase activity involved in mitotic cell cycle | 5.47E-05 |
| apoptotic process | 0.0043709 | protein ubiquitination | 0.0002161 | TRIF-dependent toll-like receptor signaling pathway | 6.31E-05 |
| innate immune response | 0.0049936 | mitotic nuclear envelope disassembly | 0.0002949 | innate immune response | 6.60E-05 |
| ligase activity | 0.0056786 | in utero embryonic development | 0.0003044 | stress-activated MAPK cascade | 8.10E-05 |
| MyD88-independent toll-like receptor signaling pathway | 0.0056831 | toll-like receptor 9 signaling pathway | 0.0004619 | apoptotic signaling pathway | 9.00E-05 |
| G1/S transition of mitotic cell cycle | 0.0058884 | vacuole | 0.0004619 | immune system process | 9.00E-05 |
| leukocyte migration | 0.0063452 | cell junction organization | 0.000528 | toll-like receptor TLR1:TLR2 signaling pathway | 0.0001072 |
| signal transduction | 0.0064582 | protein N-linked glycosylation via asparagine | 0.0005862 | toll-like receptor TLR6:TLR2 signaling pathway | 0.0001072 |
| chromatin organization | 0.007279 | hexose transport | 0.0007945 | cytoskeleton organization | 0.0001073 |
| protein ubiquitination | 0.0073374 | positive regulation of viral transcription | 0.0008439 | toll-like receptor 9 signaling pathway | 0.000141 |
| axon guidance | 0.0073845 | endoplasmic reticulum unfolded protein response | 0.0008613 | toll-like receptor 5 signaling pathway | 0.0002261 |
| transforming growth factor beta receptor signaling pathway | 0.0078036 | toll-like receptor 10 signaling pathway | 0.0009534 | MyD88-independent toll-like receptor signaling pathway | 0.000241 |
| negative regulation of transforming growth factor beta receptor signaling pathway | 0.0087652 | TRIF-dependent toll-like receptor signaling pathway | 0.0009534 | nuclear-transcribed mRNA catabolic process, nonsense-mediated decay | 0.0002411 |
| protein autophosphorylation | 0.0087652 | negative regulation of transcription from RNA polymerase II promoter | 0.0013112 | DNA damage response, signal transduction by p53 class mediator resulting in cell cycle arrest | 0.0004868 |
| insulin-like growth factor receptor signaling pathway | 0.0095186 | regulation of glucose transport | 0.0013196 | ubiquitin protein ligase binding | 0.0005857 |
| protein polyubiquitination | 0.0099917 | stress-activated MAPK cascade | 0.0013924 | negative regulation of cell proliferation | 0.0005866 |
| toll-like receptor 2 signaling pathway | 0.0101885 | transcription factor binding | 0.0014822 | negative regulation of ubiquitin-protein ligase activity involved in mitotic cell cycle | 0.0006446 |
| transcription from RNA polymerase II promoter | 0.0111266 | generation of precursor metabolites and energy | 0.0016582 | cell proliferation | 0.0007345 |
| insulin receptor signaling pathway | 0.0117489 | regulation of transcription from RNA polymerase II promoter in response to hypoxia | 0.0017164 | anaphase-promoting complex-dependent proteasomal ubiquitin-dependent protein catabolic process | 0.000787 |
| toll-like receptor 3 signaling pathway | 0.0132775 | toll-like receptor TLR1:TLR2 signaling pathway | 0.0017424 | post-translational protein modification | 0.0007911 |
| intrinsic apoptotic signaling pathway | 0.017449 | toll-like receptor TLR6:TLR2 signaling pathway | 0.0017424 | protein ubiquitination | 0.0008005 |
| cellular response to hypoxia | 0.0183045 | protein polyubiquitination | 0.0017831 | toll-like receptor 3 signaling pathway | 0.001065 |
| transcription corepressor activity | 0.0183045 | toll-like receptor signaling pathway | 0.0023585 | translation factor activity, nucleic acid binding | 0.0011984 |
| toll-like receptor 4 signaling pathway | 0.0201607 | JAK-STAT cascade involved in growth hormone signaling pathway | 0.0026065 | transcription corepressor activity | 0.0013639 |
| cell proliferation | 0.0244248 | toll-like receptor 3 signaling pathway | 0.0026928 | endoplasmic reticulum unfolded protein response | 0.0014545 |
| endosomal transport | 0.0251235 | inositol phosphate metabolic process | 0.0027095 | nuclear-transcribed mRNA poly(A) tail shortening | 0.0014615 |
| post-translational protein modification | 0.0267217 | MyD88-independent toll-like receptor signaling pathway | 0.0027553 | cellular component disassembly involved in execution phase of apoptosis | 0.0026458 |
| in utero embryonic development | 0.0267217 | cellular component movement | 0.0029375 | toll-like receptor 2 signaling pathway | 0.0026458 |
| activation of MAPKK activity | 0.0298357 | toll-like receptor 5 signaling pathway | 0.0030119 | vesicle-mediated transport | 0.0026458 |
| protein N-linked glycosylation via asparagine | 0.0316029 | regulation of ubiquitin-protein ligase activity involved in mitotic cell cycle | 0.0037016 | protein N-linked glycosylation via asparagine | 0.0028133 |
| mitotic nuclear envelope disassembly | 0.039063 | regulation of transcription involved in G1/S transition of mitotic cell cycle | 0.004974 | ligase activity | 0.0034863 |
| core promoter sequence-specific DNA binding | 0.039825 | cell projection | 0.004974 | toll-like receptor signaling pathway | 0.0036397 |
| ubiquitin binding | 0.039825 | small GTPase binding | 0.0049825 | generation of precursor metabolites and energy | 0.0036946 |
| SMAD binding | 0.039825 | cell proliferation | 0.0049825 | negative regulation of transcription, DNA-templated | 0.0037111 |
| positive regulation of apoptotic process | 0.0413135 | type I interferon signaling pathway | 0.0052839 | toll-like receptor 4 signaling pathway | 0.0042186 |
| placenta blood vessel development | 0.0418532 | chromatin organization | 0.0056225 | cytoplasmic stress granule | 0.0046276 |
| apoptotic signaling pathway | 0.0431522 | regulation of cell cycle | 0.0056225 | cellular component movement | 0.0046276 |
| transmembrane receptor protein tyrosine phosphatase signaling pathway | 0.0493156 | cytoskeleton organization | 0.0056441 | in utero embryonic development | 0.0047173 |
| plus-end-directed vesicle transport along microtubule | 0.0493156 | cellular response to hypoxia | 0.0057606 | RNA splicing | 0.0048056 |
| translation factor activity, nucleic acid binding | 0.0493156 | toll-like receptor 2 signaling pathway | 0.0061739 | activation of MAPKK activity | 0.0049221 |
| peptidyl-serine phosphorylation | 0.0493156 | mRNA splicing, via spliceosome | 0.0068219 | phosphatidylinositol biosynthetic process | 0.0061357 |
| extracellular matrix disassembly | 0.0496076 | transcription corepressor activity | 0.0080814 | negative regulation of transcription from RNA polymerase II promoter | 0.0064566 |
|  |  | DNA strand elongation involved in DNA replication | 0.0086338 | platelet activation | 0.0068587 |
|  |  | peptidyl-threonine phosphorylation | 0.0086338 | mRNA 3'-end processing | 0.0069928 |
|  |  | activation of MAPKK activity | 0.0086727 | energy reserve metabolic process | 0.0074111 |
|  |  | cytoskeleton-dependent intracellular transport | 0.0090845 | transcription factor binding | 0.0074111 |
|  |  | positive regulation of ubiquitin-protein ligase activity involved in mitotic cell cycle | 0.0094268 | protein maturation | 0.0094508 |
|  |  | extracellular matrix disassembly | 0.0111126 | histone acetyltransferase binding | 0.0113862 |
|  |  | cytokine-mediated signaling pathway | 0.0118007 | viral life cycle | 0.0113862 |
|  |  | platelet degranulation | 0.0122704 | negative regulation of transforming growth factor beta receptor signaling pathway | 0.011776 |
|  |  | micro-ribonucleoprotein complex | 0.0127074 | negative regulation of apoptotic process | 0.0122301 |
|  |  | DNA damage response, signal transduction by p53 class mediator resulting in cell cycle arrest | 0.0127074 | cell junction organization | 0.0127164 |
|  |  | ubiquitin protein ligase binding | 0.0133312 | vacuolar transport | 0.0128875 |
|  |  | anaphase-promoting complex-dependent proteasomal ubiquitin-dependent protein catabolic process | 0.0156295 | regulation of transcription from RNA polymerase II promoter in response to hypoxia | 0.013455 |
|  |  | antigen processing and presentation of exogenous peptide antigen via MHC class II | 0.0174441 | ubiquitin-specific protease activity | 0.0136426 |
|  |  | negative regulation of transforming growth factor beta receptor signaling pathway | 0.0174441 | ubiquitin-protein transferase activity | 0.0138925 |
|  |  | ligase activity | 0.0198438 | nucleolus | 0.0149183 |
|  |  | viral life cycle | 0.020418 | transcription from RNA polymerase II promoter | 0.0155527 |
|  |  | negative regulation of translation involved in gene silencing by miRNA | 0.0208043 | platelet degranulation | 0.0166341 |
|  |  | endosome | 0.0209651 | cell leading edge | 0.0191327 |
|  |  | toll-like receptor 4 signaling pathway | 0.0229523 | SMAD binding | 0.0196232 |
|  |  | kinase activity | 0.0272956 | PML body | 0.0198082 |
|  |  | cytoplasmic stress granule | 0.0276663 | 'de novo' posttranslational protein folding | 0.0214919 |
|  |  | regulation of RNA biosynthetic process | 0.0317661 | anatomical structure morphogenesis | 0.0230941 |
|  |  | axon guidance | 0.0319822 | negative regulation of translation | 0.0237404 |
|  |  | regulation of circadian rhythm | 0.0319849 | negative regulation of translation involved in gene silencing by miRNA | 0.0250534 |
|  |  | protein import into nucleus | 0.0319849 | meiotic cell cycle | 0.0272329 |
|  |  | SMAD binding | 0.0320169 | regulation of transcription involved in G1/S transition of mitotic cell cycle | 0.0273573 |
|  |  | nuclear-transcribed mRNA catabolic process, nonsense-mediated decay | 0.0343495 | glucose transport | 0.0282332 |
|  |  | ubiquitin-specific protease activity | 0.0357463 | positive regulation of apoptotic process | 0.0296432 |
|  |  | ubiquitin-protein transferase activity | 0.0366159 | cell projection | 0.0299684 |
|  |  | transcription elongation from RNA polymerase II promoter | 0.0369905 | regulation of fatty acid biosynthetic process | 0.030611 |
|  |  | microtubule cytoskeleton | 0.0373194 | RISC complex | 0.034423 |
|  |  | cellular component disassembly involved in execution phase of apoptosis | 0.0382097 | cellular protein localization | 0.0346949 |
|  |  | protein maturation | 0.0384273 | vacuole | 0.0365334 |
|  |  | cellular lipid metabolic process | 0.0412976 | protein K48-linked deubiquitination | 0.0369492 |
|  |  | cell junction assembly | 0.0426486 | ribonucleoprotein complex assembly | 0.0389704 |
|  |  | negative regulation of cell proliferation | 0.0448955 | microtubule cytoskeleton | 0.0389704 |
|  |  | nitric oxide metabolic process | 0.0499141 | androgen receptor binding | 0.0395648 |
|  |  |  |  | mRNA splicing, via spliceosome | 0.0409329 |
|  |  |  |  | PcG protein complex | 0.0410729 |
|  |  |  |  | MyD88-dependent toll-like receptor signaling pathway | 0.0414641 |
|  |  |  |  | proteasome-mediated ubiquitin-dependent protein catabolic process | 0.0422648 |
|  |  |  |  | transcriptional repressor complex | 0.0443474 |
|  |  |  |  | establishment or maintenance of cell polarity | 0.045946 |
|  |  |  |  | focal adhesion | 0.0490689 |
